# The effect of short-chain fatty acids on human monocyte-derived dendritic cells

**DOI:** 10.1038/srep16148

**Published:** 2015-11-06

**Authors:** Claudia Nastasi, Marco Candela, Charlotte Menné Bonefeld, Carsten Geisler, Morten Hansen, Thorbjørn Krejsgaard, Elena Biagi, Mads Hald Andersen, Patrizia Brigidi, Niels Ødum, Thomas Litman, Anders Woetmann

**Affiliations:** 1 Department of Immunology and Microbiology, University of Copenhagen, Denmark; 2 Department of Pharmacy and Biotechnology, University of Bologna, Italy; 3 Center for Cancer Immune Therapy (CCIT), Department of Hematology, Copenhagen University Hospital, Herlev, Denmark; 4 Translational Research, LEO Pharma, Denmark

## Abstract

The gut microbiota is essential for human health and plays an important role in the pathogenesis of several diseases. Short-chain fatty acids (SCFA), such as acetate, butyrate and propionate, are end-products of microbial fermentation of macronutrients that distribute systemically via the blood. The aim of this study was to investigate the transcriptional response of immature and LPS-matured human monocyte-derived DC to SCFA. Our data revealed distinct effects exerted by each individual SCFA on gene expression in human monocyte-derived DC, especially in the mature ones. Acetate only exerted negligible effects, while both butyrate and propionate strongly modulated gene expression in both immature and mature human monocyte-derived DC. An Ingenuity pathway analysis based on the differentially expressed genes suggested that propionate and butyrate modulate leukocyte trafficking, as SCFA strongly reduced the release of several pro-inflammatory chemokines including CCL3, CCL4, CCL5, CXCL9, CXCL10, and CXCL11. Additionally, butyrate and propionate inhibited the expression of lipopolysaccharide (LPS)-induced cytokines such as IL-6 and IL-12p40 showing a strong anti-inflammatory effect. This work illustrates that bacterial metabolites far from the site of their production can differentially modulate the inflammatory response and generally provides new insights into host-microbiome interactions.

The gut microbiota is essential for several aspects of human health and is involved in the pathogenesis or as protection factor for several human diseases, such as obesity, inflammatory bowel disease, type 1–2 diabetes, atopic disease, acute graft-versus host disease^1^,^2^,^3^,^4^. Recent studies highlight the profound effect of fiber-rich diet on the metabolism and composition of the gut microbiota, with profound feedback consequences on the host immunological pathways^5^,^6^. However, most of the mechanisms of host-microbiota interactions remain obscure. Dietary fibers have been linked with a lower risk of death from a range of conditions, including cardiovascular disease, infections and cancers^7^. It seems clear that making fiber-rich food choices more often may provide significant health benefits but, however, little is known about the mechanisms. Dietary fibers are complex carbohydrates that can be fermented by fibrolythic components of the gut microbiota, generating physiologically active metabolites. Short-chain fatty acids (SCFA), such as acetate, butyrate and propionate, are among the most abundant of these dietary metabolites released in the proximal colon at high concentrations (70–140 mM) while lower concentrations are found in the distal colon (20–70 mM) and in the distal ileum (20–40 mM). The molar ratio of acetate, propionate, and butyrate production in the colon is approximately 60:25:15, respectively, but it varies depending on diet, site of fermentation, and microbiota composition^8^,^9^. Notably, SCFA are not restricted to the intestinal lumen but are readily absorbed by colonocytes, released into the bloodstream and then reach the liver through the portal vein. From here SCFA are then released systematically to the peripheral venous system at even lower concentrations^10^. Experiments carried out on mice models allowed dissection of the multifactorial role of these metabolites on the host immunological phenotype. SCFA have been shown to modulate several cell signaling pathways in immune cells^11^,^12^,^13^, indicating that intestinal microbiota shape the immune system through their metabolites, and hence that SCFA are key mediators for the host-microbe mutualism. Indeed, the exacerbated inflammatory reactions observed in germ-free mouse models of disease are likely to relate in part to the absence of SCFA in the gut, blood, or tissues^14^,^15^. SCFA exert their effects through G-protein-coupled receptors (GPCRs). The major GPCRs activated by SCFA are GPR43 (FFAR2) and GPR41 (FFAR3), and GPR109A (HCAR2) for both murine and human immune cells^16^,^17^,^18^,^19^,^20^,^21^. However, the expression pattern of SCFA receptors in human DC has been poorly investigated, although expression of GPR41 and GPR43 mRNA in human DC has been described^16^,^18^.

DC are professional antigen-presenting cells within the immune system and once activated they play a pivotal role in attracting and priming T-cells. Recently, propionate was shown to affect mouse dendritic cells (DC) and macrophage biology in the bone marrow and affect T helper 2 (Th2) cell responses in the airway^22^ confirming the important role of SCFA on the mouse immune system. However, little is known about the effect of SCFA on human DC. The experimental use of human primary DC is limited by their scarcity in peripheral blood (<1% of peripheral blood mononuclear cells (PBMC)), so to circumvent this, monocyte-derived DC *in vitro* are commonly employed as a pragmatic model^23^,^24^.

The aim of this study was to investigate the gene transcription modulation by Affymetrix GeneChip and potential pathways in human immature and mature human monocyte-derived DC after exposure to acetate, butyrate, or propionate. The results revealed distinct transcriptional responses elicited by these bacterial metabolites indicating that butyrate and propionate exerts important immunomodulatory roles by down-regulating genes involved in inflammation and immune cell trafficking.

## Results

### SCFA receptor expression by human monocyte-derived DC

**Figure 1:**
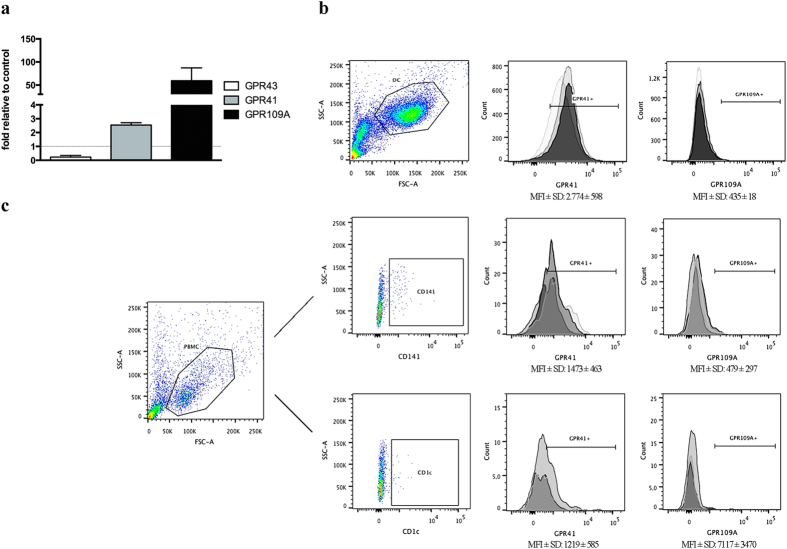
Expression of SCFA receptors by human monocyte-derived DC and primary CD141 + and CD1c + DC. (**a**) qPCR for SCFAs receptors on monocyte-derived DC compared to MCF-7 cell line as internal control. Shown are the averages ± standard deviations (SD) (n = 3). (**b**) Flow cytometry analysis of GPR41 and GPR109A surface expression on monocyte-derived DC and (**c**) primary CD141 + and CD1c + DC. Mean fluorescence intensity (MFI) averages ± standard deviations (SD) (n = 3) are reported below each histogram. Gates for GPR41- or GPR190A-positive cells in both figures (**b,c**) has been placed according to their respective isotype control. The different shades of grey represent cells from three different donors used for the experiments.

To determine the expression pattern of SCFA receptors on human monocyte-derived DC, we performed qPCR assays for the main SCFA receptors genes such as GPR43, GPR41, and GPR109A. MCF-7 cells were used as positive control as they have previously been reported to express both GPR43 and GPR41^25^. As shown in Fig. 1a we found that human monocyte-derived DC expresses GPR43 and GPR41 mRNA at lower and higher level, respectively, compared to the positive control cell line MCF-7. In addition, we observed very high expression levels of GPR109A mRNA in human monocyte-derived DC. Furthermore, we investigated the surface expression of GPR41 and GPR109A on human monocyte-derived DC by flow cytometry. As shown in Fig. 1b, we used cells from three different donors, (each donor represented by different shades of grey), to confirm that both receptors are expressed, albeit we observed that surface proteins expression of GPR41 was higher than GPR109A.

Furthermore, we examined the expression of SCFA receptors on primary human CD1c+ and CD141 + DC by flow cytometry. We found that both subsets of DC express GPR41 and GPR109 on the surface of cells from three different donors (Fig. 1c, each donor represented by a different shade of grey). Taken together, this indicates that human monocyte-derived DC, like primary human CD1c+ and CD141+ DC, has the ability to respond to SCFA through GPR41 and GPR109A.

### DC exposure to SCFA and impact on maturation markers

Monocytes isolated from three different healthy donors were used to generate both im-DC and m-DC.

Then im-DC were treated with 1 mM (physiological concentration) of sodium acetate (im-DC_A), sodium butyrate (im-DC_B), or sodium propionate (im-DC_P) for the last 24 h of culture. In parallel, in order to explore how SCFA modulates the lipopolysaccharide (LPS) -induced maturation of human monocyte-derived DC, we exposed im-DC to the LPS, at the same time of the SCFA addition. So, after 24 h, we obtained the following groups of treatment named: m-DC_A, m-DC_B, and m-DC_P.

Im-DC SCFA-untreated (im-DC) and m-DC SCFA-untreated (m-DC) were used as controls for the immature and mature state, respectively. Treatment with 1 mM of each SCFA did not change the percentage of apoptotic cells (untreated monocyte-derived DC 1.4%, acetate 1.1%, propionate 1.0%, and butyrate 1.8% respectively).

**Figure 2:**
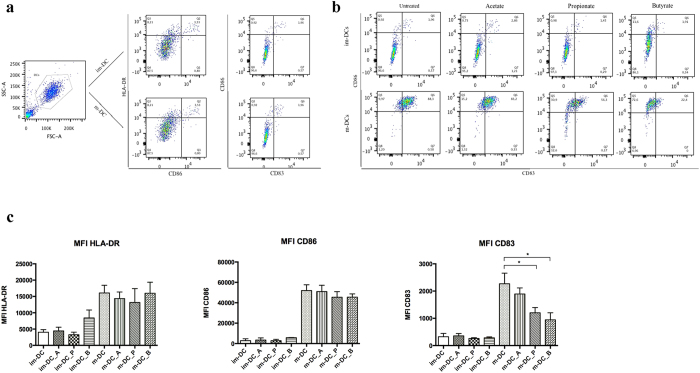
(**a**) Flow cytometry DC gating and SCFA effect. The figure is representative of the gating strategy for DC for HLA-DR, CD83, and CD86 markers for both monocyte-derived im-DC and m-DC. (**b**) A representative example of SCFA effect on im-DC and m-DC expression of CD86 and CD83 markers by flow cytometry. (**c**) Effect of SCFA on DC maturation markers. Numbers calculated represented by flow cytometry indicate the mean fluorescence intensity (MFI) of each sample. Shown are the averages ± standard deviations (SD) (n = 3); Mann-Whitney U t-test, *P < 0.05.

In order to validate the DC’s maturation stage flow cytometry analysis was performed with particular attention to the surface expression of maturation markers such as HLA-DR, CD83 and CD86^24^,^26^,^27^. As expected, their expression upon the membrane was increased after the addition of LPS to the culturing media (Fig. 2a). Next we investigate the impact of SCFA on human monocyte-derived DC maturation makers. Interestingly, the presence of SCFA did not affect the expression of either HLA-DR or CD86, whereas the LPS-induced expression of CD83 was significantly reduced by the exposure to both propionate and butyrate when compared to normally matured DC (Fig. 2b,c).

### SCFA effect on immature and mature DC

**Figure 3:**
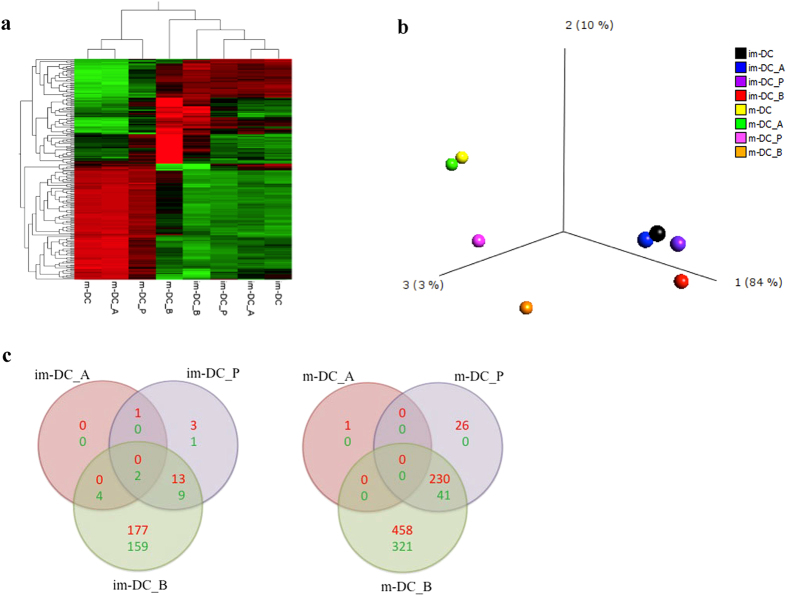
(**a**) Heat-map and unsupervised hierarchical clustering based on the top 200 differentially expressed genes (DEG). (**b**) Principal component analysis (PCA) sample plot based on the 737 most variable genes across experiments. Primary clustering is seen according to im-/m-dendritic cells (DC). The samples are colored according to treatment with either acetate (**a**), propionate (P), or butyrate (**b**). Each dot represents one pooled sample from three donors. (**c**) Venn diagrams showing the number of overlapping up-(red) and down-(green) regulated genes by im-DC and m-DC after exposure to A (acetate), P (propionate), or B (butyrate).

Comparing untreated im-DC to untreated m-DC revealed 1752 differentially expressed genes (DEG) (cut off criteria: >2-fold change, p < 0.05) emerged by Affymetrix GeneChip. Exposure to SCFA resulted in significant modulation of the m-DC gene expression but only in a moderate effect on the im-DC gene expression (Fig. 3a).

Visualization of the samples by principal component analysis (PCA) further supported the specific and stronger effect of butyrate and propionate on the m-DC. The same behavior was observed for im-DC, with an overall lower variation in the expression profile. In contrast, the effect of acetate treatment is similar to the one shown by untreated controls (both for im-DC and m-DC) (Fig. 3b). Indeed, the transcriptome analysis revealed that butyrate and propionate, more than acetate, elicit a specific response by both treated im-DC, and m-DC; the general effect exerted by SCFA can be ranked as: acetate < propionate < butyrate. Acetate only induced minor changes in gene expression (8 DEG) in im-DC, and none in m-DC.

Further, acetate seems not to share many affected genes with either butyrate (none and 4, up- and down-regulated genes, respectively) or propionate (1 and 0, respectively) in immature DC. Further, acetate has no effect on mature DC not sharing any DEG in common with butyrate and propionate. Propionate affects few genes (13 up-, and 9 down- regulated) while butyrate shows the biggest effect—up-regulating 177 and down-regulating 159 genes—on im-DC. Furthermore, butyrate and propionate share 230 up- and 41 down-regulated genes in m-DC and, again, butyrate exerts an even more profound effect on m-DC, up- and down-regulating 458 and 321 genes, respectively (Fig. 3c).

Ingenuity pathway analysis (IPA) based on DEGs identified no significant networks or pathways involved by acetate treatment emphasizing the effect exerted by butyrate and propionate. In fact, IPA analysis revealed “granulocyte adhesion and diapedesis” as one of the top canonical pathways following exposure to butyrate, for both im-DC and m-DC; similar findings apply to the effect of propionate, albeit only on m-DC. Indeed among the down-regulated genes are found several chemokines that have been affected by butyrate and propionate, especially in the mature stage of DC (Table 1). The top molecules down-regulated in the m-DC cells after butyrate treatment when compared to the untreated control are CXCL9 (log2-fold change) (−5.4), CXCL10 (−5.1), CXCL11 (−5.3), CCL19 (−4.0) and the top molecules that are down-regulated by propionate treatment includes CXCL9 (−2.0), CCL19 (−1.7), IL-6 (−2.1), IL12B (−1.8) (S1) indicating an overall immunomodulatory effect exerted by both of these SCFA. Among the genes up-regulated by butyrate in both im-DC and m-DC we noticed ALDH1A1 (aldehyde dehydrogenase 1 family, member 1), that codes for an enzyme involved in metabolizing retinoic acid and that exerts an immunomodulatory role in mice^28^.

### Immunomodulatory effects of butyrate and propionate on m-DC

**Figure 4:**
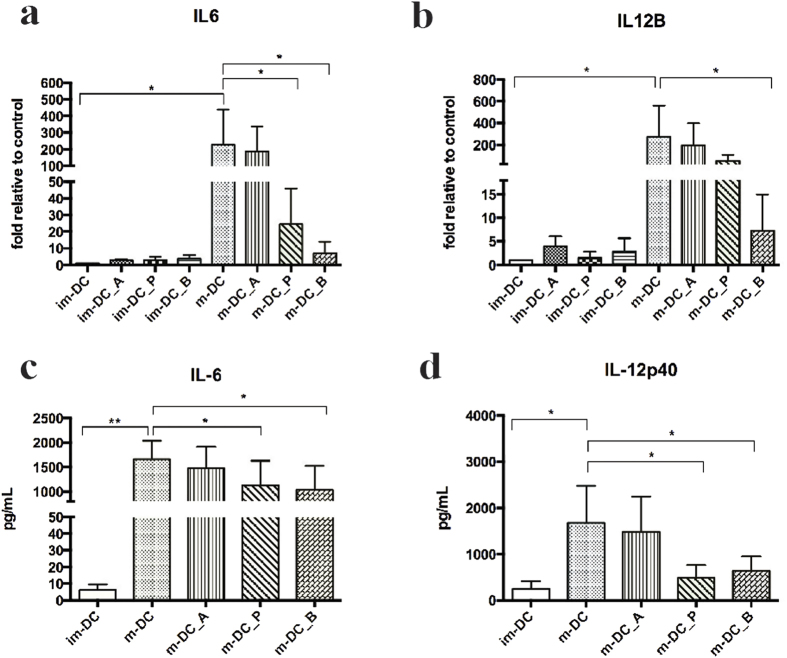
IL6 and IL12B qPCR ((**a,b**) respectively) and IL-6 and IL-21p40 ELISA ((**c,d**) respectively) on monocyte-derived m-DC and im-DC treated with acetate, propionate, and butyrate. Shown are the averages ± standard deviations (SD) (n = 3); Mann-Whitney U t-test p values: *P ≤ 0.05; **P ≤ 0.01.

The transcriptome analysis revealed that butyrate and propionate exert a strong immunomodulatory effect on mature DC. For LPS-treated DC the butyrate effect is most pronounced in normalizing the gene expression levels back to those observed in the PBS-treated DC down-regulating the CXCL9, CXCL10, CXCL11, and IL12B genes. The propionate effect is similar albeit weaker than butyrate and also decreases the expression of CXCL9, IL6 and IL12B. To confirm the microarray data we performed qPCR for IL6 and IL12B in parallel with ELISA assays for secreted IL-6 and IL12p40 (IL12B) protein. Both butyrate and propionate significantly reduced LPS-induced IL6 mRNA expression (P < 0.05, Fig. 4a), whereas the effect on IL12B gene expression was significant (P < 0.05) only after butyrate treatment but not for propionate, as shown in Fig. 4b. The effect on LPS-induced IL-6 was confirmed by ELISA for IL-6 release by mature DC exposed to butyrate and propionate as protein levels were significantly reduced (P < 0.05, respectively) (Fig. 4c). The effect of SCFA on LPS-induced production of IL-12p40 by human monocyte derived DC was likewise confirmed by ELISA. As shown in Fig. 4d, both propionate and butyrate significantly reduced the level of secreted IL-12p40 protein (P < 0.05, respectively).

**Figure 5:**
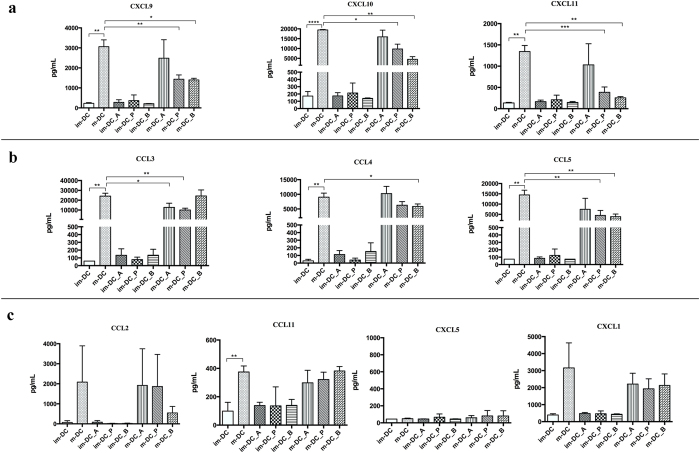
Chemokine patterns by im-DC and m-DC treated with acetate, propionate, and butyrate. Shown are the averages ± standard deviations (SD) (n = 3); Unpaired t test with Welch’s correction, *P < 0.05; **P < 0.01; ***P < 0.001; ****P < 0.0001.

Furthermore, we measured the chemokines secreted into the media through LEGENDPlex™ array confirming a significant reduction of CXCL9, CXCL10, and CXCL11 production after butyrate and propionate exposure by m-DC (Fig. 5a).

In order to further investigate the effect of SCFA on DC chemokine patterns we measured ten chemokines by LEGENDPlex™ array. This analysis revealed that im-DC are not responsive to the addition of SCFA in contrast to mature DC whose chemokine production changes specifically depending on what SCFA is added into the media. As mentioned above, butyrate and propionate, but not acetate, significantly reduce the secretion of CXCL9, −10, −11 by m-DC (Fig. 5a). Further, CCL3 release is significantly reduced by acetate and propionate, but not by butyrate; CCL4 is reduced only by butyrate but not by acetate neither by propionate; CCL5 production decreases after butyrate and propionate exposure but is not modified by acetate (Fig. 5b). CCL2, CCL11, CXCL11 are not significantly affected by SCFA and CXCL5 is not produced at all by m-DC (Fig. 5c).

## Discussion

Human beings have been recently reconsidered as super-organisms in co-evolution with an immense microbial community living in the gastrointestinal tract that is essential for the development, education and functionality of our immune system^29^. Dendritic cells behave as sentinels of the immune system and their function is to sample antigen in inflamed tissue and migrate to the local lymph nodes where the DC besides the presentation of these antigens to T naive cells also produce cytokines, thus influencing the polarization into different T-helper-cell subsets e.g. Th1, Th2 or Th17. In this study we hypothesized that SCFA, secreted into the gut and distributed through blood to tissues, have immunomodulatory effects on human dendritic cells that, as APCs, have the ability to shape the immune system’s response.

Immune cells, including DC, use specific receptors to sense and respond to bacterial metabolites. According to the literature, one largely used mechanism is via metabolite-sensing GPCRs such as, GPR43, GPR41, and GPR109A, all of which can act as receptors, with different specificity and affinity, for SCFA^16^,^18^,^20^,^21^,^30^. Indeed, many of the actions related to SCFA, and linked to gut homeostasis, have been ascribed to GPR43 and GPR109A that are both expressed by inflammatory leukocytes (such as neutrophils and macrophages) and by T regulatory cells (Tregs)^13^,^28^. Recently, GPR109A has emerged as a major regulator of gut immune homeostasis^28^ binding not only the SCFA butyrate but also the tryptophan metabolite nicotinic acid, whose anti-inflammatory properties are well known^31^,^32^. However, SCFA effects are not restricted to GPR43 and GPR109A, given that a recent study established a role for propionate and the SCFA receptor GPR41 in the generation of macrophage and DC precursors^22^. As shown in Fig. 1a, we observed that GPR41 and GPR109A are both expressed by human monocyte-derived DC, whereas GPR43 is only weakly expressed. Surface expression of GPR41 and GPR109A was confirmed by flow cytometry (Fig. 1b), indicating that both these receptors may be important for the SCFA induced signal transduction.

We also examined the expression of GPR41 and GPR109A on human primary CD1c+ and CD141+ DC by flow cytometry, and found the both subset of cells express the receptors on the surface, indicating that primary human DC has the ability to respond to SCFA comparable to human monocyte-derived DC.

The transcriptomic analysis revealed that butyrate and propionate, more than acetate, elicit a specific response by human DC. Furthermore, butyrate and propionate share the largest number of affected host dendritic cell genes. This is supported by Lukovac *et al.*, who reported a similar trend in the effect of propionate and butyrate on epithelia cells in a *in vitro* murine gut organoid model^33^. In particular, among these observations we focused on the potential immunomodulatory effect exerted by SCFA on the lipopolysaccharide (LPS) -induced maturation of human monocyte-derived DC, previously reported on another type of APC cells such as bone marrow-derived macrophages in a murine model^12^. It is known that macrophages, once activated by LPS, have the important role to prime and enhance the inflammasome producing large amounts of CCL2, TNF-α, IL-12p40 and IL-6. Additionally, it has been shown that mainly butyrate suppresses the production of those inflammatory mediators by monocytes and macrophages^16^,^34^ and that it seems to enhance the release of the anti-inflammatory cytokine IL-10^35^. In accord with these results, we observed that among the SCFA analyzed, butyrate and propionate play a crucial role in modulating immune responses in human mature dendritic cells. In particular, in our study, propionate shows the ability to significantly reduce IL-6 expression and protein release, as well as butyrate. In addition, both butyrate and propionate reduced the LPS-induced gene expression and protein production of IL-12B (IL12p40), a shared component of both IL-12 and IL-23 formation^36^. This latter data lead us to hypothesize that both SCFA, compromising the IL-12 and IL-23 production, could shape the naïve T-cell polarization by reducing the pro-inflammatory Th1 and Th17 phenotypes and therefore shifting the balance towards anti-inflammatory populations such as Tregs, as already shown in mice^11^. In addition, it also appears that the activity of butyrate and propionate is selective because these SCFA affect primary LPS response genes such as TNF-α family genes and CCL2, while other LPS response genes such as HLA-DR, CD86, IL-1A, IL-1B, are unaffected or even up-regulated (Fig. 2c and Supplementary 1).

Another aspect of our study was to explore how the DC chemokine pattern was influenced by SCFA taken that the chemokine production is instrumental for DC to regulate the recruitment of different cell types for both the afferent and efferent limb of the immune response^37^. In addition to their chemotactic effect on neutrophils, it is known that SCFA also modulate production and release of chemokines and expression of adhesion molecules in neutrophils and endothelial cells^38^,^39^, which may be relevant to their effect on leukocyte recruitment. We observed that in the first 24h after exposure to SCFA each of them showed a distinctive effect; acetate reduces the release of CCL3, butyrate decreases CCL4, CCL5 and CXCL9, −10, −11 and propionate CCL3, CCL5 and CXCL9, −10, −11, both in terms of gene expression and protein production. Previously, Sallusto *et al.* showed that inflammatory chemokines, such as CCL3, CCL4, and CCL5, can be induced during maturation/activation of human monocyte-derived DC^37^ and, together with the other pro-inflammatory chemokines CXCL9, CXCL10, CXCL11, our results strongly support the notion that SCFA exert an immunomodulatory effect *per se* directly on DC. It is likely that inflammatory chemokines regulate cell traffic within secondary lymphoid tissues and, thus, it may influence T cell development during antigen recognition^40^. Our data clarify that among the three SCFA studied, butyrate and propionate exert the main immunomodulatory role directly influencing the gene expression profile of DC. These findings open a new perspective about the SCFA ability to shape the inflammatory response through DC, regulating leukocyte polarization and recruitment.

## Materials and Methods

### Human monocyte-derived DC generation and culture

DC was generated from peripheral blood mononuclear cells (PBMC) isolated from buffy coats obtained from anonymous healthy blood donors (n = 3). Written informed consent was obtained from blood donors at the Department of Clinical Immunology, University Hospital Rigshospitalet, Copenhagen and used without the possibility to identify case specific information. The ethical committee, Region H, The Capital Region of Denmark, approved the use of these buffy coats for research. The research was carried out in accordance with the approved guidelines. The mononuclear cells were separated by Ficoll-Hypaque density gradient centrifugation and subsequently CD14+ monocytes were isolated from PBMCs by positive selection using magnetic beads (Miltenyi Biotec, Germany), according to the manufacturer’s instructions. Monocytes were cultured at 37 °C in 5% CO_2_ in media supplemented with 10% fetal bovine serum (FBS, Gibco) GM-CSF and IL-4 (50 ng/ml both, PeproTech) for 7 days to generate im-DC and m-DC, after LPS (100 ng/ml, *E.coli* 055:B5, Sigma-Aldrich) stimulation for the last 24h.

### Human primary CD141+ and CD1C+ analysis

PBMCs were isolated from buffy coats (n = 3) as described above and primary dendritic cells CD141+ and CD1C+ were analyzed by flow cytometry in triplicate.

### SCFA treatments

Before SCFA treatment was performed, a 7-AAD (Sigma-Aldrich) assay was applied to determine the SCFA concentration that does not affect DC viability. Based on the 7-AAD assay, 1mM was chosen for each SCFA treatment as representative of human gut physiologic concentration. For both im-DC and m-DC the last 24h were used for the single exposure to acetate, propionate or butyrate (Sigma-Aldrich).

### 7-Aminoactinomycin apoptosis assay

Cells were stained with 7-aminoactinomycin, and subjected to flow cytometry analysis as described elsewhere^41^.

### Flow cytometry analysis

Cell staining was performed by LSR Fortessa (BD) at the CFFC (Core Facility for Flow Cytometry, Faculty of Health and Medical Sciences, University of Copenhagen). Anti-human HLA-DR (PE, BD, clone L243), anti-human CD83 (BV421, Biolegend, clone HB15e) and anti-human CD86 (APC, Biolegend, clone IT2.2), anti-human CD141 (BV421, BD, clone 1A4), anti-human CD1c (BB515, BD, clone F10/21A3), anti-human GPR41 (APC, LSBio), anti-human GPR109A (APC, R&D), and all the respective isotypes were used (all purchased by BD or R&D). FACS data were analyzed by FlowJo v7.0.

### LEGENDPlex and ELISA arrays

IL-6 and IL-12p40 production was measured in the supernatants of DC by ELISA using R&D kits. For simultaneous quantification of chemokines we applied the bead-based multiplex assay LEGENDPlex (BioLegend) and analyzed by LSRFortessa (BD) at the CFFC.

### RNA isolation and expression profiling

RNA was isolated and purified using the RNA was isolated and purified using the RNeasy kit (Quiagen) according to the manufacturer’s instructions, then quantified using a spectrophotometer (NanoDrop, Wilmington, DE). Equal amounts of RNA derived from the three donors were pooled, each treatment with its own corresponding control. Sample labeling and microarray hybridization were performed according to the manufacturer’s instructions (Affymetrix).

Global gene expression analysis was conducted using Affymetrix GeneChip® Human Transcriptome Array HTA 2.0 covering > 285.000 full-length transcripts (performed by AROS Applied Biotechnology A/S, Aarhus, DK). The array data were normalized using Robust Multichip Average (RMA) normalization as recommended by Bolstad *et al.*^42^. Significance of DEG (2-fold change, p < 0.05) was assessed by ANOVA, and adjusted for multiple testing by estimating false discovery rates (FDR). Data visualization, including principal component analysis (PCA), heat maps and clustering was performed in Qlucore Omics Explorer v.3.0 (Qlucore AB, Lund, Sweden). Functional analysis and network representation of DEG was performed in Ingenuity Pathway Analysis (IPA, Ingenuity® Systems).

### Real-time PCR

Total RNA was isolated using RNeasy Mini Kit (Qiagen) and cDNA was transcribed using the High Capacity cDNA Reverse Transcription Kit followed by PCR analysis using TaqMan® Gene Expression Assay method. All TaqMan probes were purchased from LifeTechnologies (GAPDH (Hs02758991_g1), GPR43 (Hs00271142_s1), GPR41 (Hs02519193_g1), GPR109A (Hs02341584_s1), IL6 (Hs00985639_m1), IL12B (Hs01011518_m1). Amplification was performed in an Mx3000P real-time thermal cycler (Stratagene) on standard settings. Data presented here was obtained from three independent experiments. Each experiment included three technical replicates. Results are presented as relative quantity to the control sample determined by the ddCt method, using GAPDH as reference gene and untreated im-DC as calibrator.

### Microarray data accession number

Raw microarray data have been deposited in the GEO database (http://www.ncbi.nlm.nih.gov/geo/) under accession no. GSE66989.

## Additional Information

**How to cite this article**: Nastasi, C. *et al.* The effect of short-chain fatty acids on human monocyte-derived dendritic cells. *Sci. Rep.* **5**, 16148; doi: 10.1038/srep16148 (2015).

## Supplementary Material

Supplementary Information

Supplementary Dataset

## Figures and Tables

**Table 1:** IPA analysis report.

Comparison	Top canonical pathway	Top diseases and bio functions	Top networks
m-DCs vs. im-DCs	Dendritic cell maturation	Immunological diseases	Cellular Movement, Hematological System Development and Function, Immune Cell Trafficking
imDCs_A vs. im-DC	No effect		
imDC_B vs. im-DC	Granulocyte adhesion and diapedesis; agranulocyte adhesion and diapedesis	Inflammatory response	Antigen Presentation, Lipid Metabolism, Small Molecule Biochemistry
imDCs_P vs. im-DCs	Eicosanoid signaling	Inflammatory response; hematological system development and function; cell-to-cell signaling and interaction	Cardiovascular Disease, Inflammatory Response, Cell-To-Cell Signaling and Interaction
mDCs_A vs. mDCs	no effect		
mDCs_B vs. mDCs	Granulocyte adhesion and diapedesis; role of Pattern Recognition Receptors in Recognition of Bacteria and Viruses; dendritic cell maturation	Cell movement; Cellular function and maintenance; hematological system development and function	DNA Replication, Recombination, and Repair, Nucleic Acid Metabolism, Small Molecule Biochemistry
mDCs_P vs. mDCs	Granulocyte adhesion and diapedesis; dendritic cell maturation; graft-versus-host disease signaling	Immunological disease; inflammatory response; cell-to-cell signaling and interaction; hematological system development and function	Cellular Function and Maintenance, Cellular Development, Hematological System Development and Function
mDCs_B vs. mDC_A	Granulocyte adhesion and diapedesis; role of Pattern Recognition Receptors in Recognition of Bacteria and Viruses; dendritic cell maturation	Inflammatory response; Cellular function and maintenance; hematological system development and function	Cell Morphology, Cellular Development, Embryonic Development
mDCs_P vs. mDC_A	Granulocyte adhesion and diapedesis; dendritic cell maturation; Communication between Innate and Adaptive Immune Cells	Immunological disease; Cell-to-cell signaling and interaction; immune cell trafficking; hematological system development	Cell-To-Cell Signaling and Interaction, Hematological System Development and Function, Immune Cell Trafficking
mDCs_P vs. mDC_B	Granulocyte adhesion and diapedesis; agranulocyte adhesion and diapedesis	Immunological disease; Cell-to-cell signaling and interaction; hematological system development	Antimicrobial Response, Inflammatory Response, Infectious Disease

The table shows the top canonical pathways, diseases and bio-functions and networks involved after SCFA exposure. Supplement 1. The top DEG according to each experimental condition. Column annotation: Gene symbol: The official gene symbol according to HUGO nomenclature. im-DC: Log2(AFU) in im-DC. m/im: The log2-ratio between m-DC and im-DC. A: The log2-ratio between acetate-treated im-DC and control im-DC. P: The log2-ratio between propionate-treated im-DC and control im-DC. B: The log2-ratio between butyrate-treated im-DC and control im-DC. Am: The log2-ratio between acetate-treated m-DC and control m-DC. Pm: The log2-ratio between propionate-treated m-DC and control m-DC. Bm: The log2-ratio between butyrate-treated m-DC and control m-DC. B/Pm: The log2-ratio between butyrate-treated m-DC and propionate-treated m-DC.
