# Supplementary material for: The effect of short-chain fatty acids on human monocyte-derived dendritic cells
Source: Sci Rep. 2015 Nov 6;5:16148. doi: 10.1038/srep16148 (PMC4635422; doi:10.1038/srep16148)
Supplement: Supplementary Information [file srep16148-s1.doc]

**The effect of short-chain fatty acids on human monocyte-derived dendritic cells**

**Claudia Nastasi,**^a^ **Marco Candela,**^b^ **Charlotte Menné Bonefeld,**^a^ **Carsten Geisler,** ^a^ **Morten Hansen,^c^ Thorbjørn Krejsgaard,**^a^ **Elena Biagi,** **^b^ Mads Hald Andersen,^c^ Patrizia Brigidi,**^b^ **Niels Ødum,**^a^ **Thomas Litman**^d^ **and Anders Woetmann**^a*^

Department of Immunology and Microbiology, University of Copenhagen, Denmark^a^; Department of Pharmacy and Biotechnology, University of Bologna, Italy^b^; Center for Cancer Immune Therapy (CCIT), Department of Hematology, Copenhagen University Hospital, Herlev, Denmark^c^; Translational Research, LEO Pharma, Denmark^d^.

*Address correspondence to: Anders Woetmann, Department of Immunology and Microbiology, Blegdamsvej 3C, University of Copenhagen, Copenhagen, Denmark. E-mail address: [awoetmann@sund.ku.dk](mailto:awoetmann@sund.ku.dk)

**Supplement 1.** The top DEG according to each experimental condition.

Column annotation:
Gene symbol: The official gene symbol according to HUGO nomenclature.

im-DC: Log2(AFU) in im-DC.
m/im: The log2-ratio between m-DC and im-DC.

A: The log2-ratio between acetate-treated im-DC and control im-DC.

P: The log2-ratio between propionate-treated im-DC and control im-DC.

B: The log2-ratio between butyrate-treated im-DC and control im-DC.

Am: The log2-ratio between acetate-treated m-DC and control m-DC.

Pm: The log2-ratio between propionate-treated m-DC and control m-DC.

Bm: The log2-ratio between butyrate-treated m-DC and control m-DC.

B/Pm: The log2-ratio between butyrate-treated m-DC and propionate-treated m-DC.
